# The Role of Biologics in Rheumatoid Arthritis: A Narrative Review

**DOI:** 10.7759/cureus.33293

**Published:** 2023-01-03

**Authors:** Jay P Patel, Nithin Kumar Konanur Srinivasa, Akshay Gande, Madatala Anusha, Hassaan Dar, Dheeraj B Baji

**Affiliations:** 1 Research, Chirayu Medical College and Hospital, Bhopal, IND; 2 Research, Bangalore Medical College and Research Institute, Bangalore, IND; 3 Research, Gandhi medical college and hospital, Secunderabad , IND; 4 Internal Medicine, Sri Devaraj Urs Medical College, Kolar, IND; 5 Research, Jinnah Medical and Dental College, Islamabad, PAK; 6 Research, All India Institute of Medical Sciences, Bhubaneswar, Bhubaneswar, IND

**Keywords:** rheumatoid arthritis, degenerative joint disease, inflammatory mediators, overview of biological drugs, intervention in rheumatoid arthritis, methotrexate, interleukin, tumor necrosis factor-alpha (tnf-α) inhibitors, systemic inflammatory and autoimmune disease, biologic agents

## Abstract

Rheumatoid arthritis (RA) is a chronic inflammatory joint disease that can cause cartilage and bone damage as well as a disability. Various cytokines play an essential role in disease formation such as tumor necrosis factor (TNF)-alpha, interleukin (IL)-1, IL-6, IL-17, and macrophages; osteoclast is also activated by the cytokines, which cause bone degradation. Early diagnosis is key to optimal therapeutic success, particularly in patients with well-characterized risk factors for poor outcomes such as high disease activity, presence of autoantibodies, and early joint damage. Treatment algorithms involve measuring disease activity with composite indices, applying a treatment-to-target strategy, and using conventional, biological, and new non-biological disease-modifying antirheumatic drugs. After the treatment target of stringent remission (or at least low disease activity) is maintained, dose reduction should be attempted. Although the prospects for most patients are now favorable, many still do not respond to current therapies. The biologics have changed the disease progression over the past few decades, such as TNF-alpha inhibitors (infliximab, etanercept, adalimumab, golimumab, certolizumab), IL-1 inhibitors (anakinra), IL-6 inhibitors (tocilizumab), CD20 inhibitors (rituximab), and cytotoxic T-lymphocyte associated antigen (CTLA)-4 inhibitors (abatacept). In treatment with biologics, only little is known if "biologic-free" remission is possible in patients with sustained remission following intensive biological therapy. Infliximab and etanercept, in the long run, develop the drug antibody. This article has reviewed the action of the cytokine on joints and biological drug's action in blocking the cytokine degradation effect, benefits of biologics, and adverse effects in the long and short term. They are also effective alone or in combination with other drugs.

## Introduction and background

Rheumatoid arthritis (RA) is a multi-etiologic, autoimmune, and chronic inflammatory disorder associated with pain, swelling, morning stiffness, and symmetrical involvement of multiple peripheral joints as destruction of joints is rarely apparent in the early stage [1]. RA is a systemic disorder usually accompanied by extra-articular manifestations such as serositis, vasculitis, Felty's syndrome, peripheral neuropathy, and pulmonary involvement in the later course of the disease [1]. On the one hand, there has been proof of RA existing in 1500 BC, with the disease manifesting itself in mummies in ancient Egypt, while on the other hand, French Surgeon Augustin Jacob Landre-Beauvais published the first report on RA in the year 1800, making it the disease of the modern era [2-5]. Studies have demonstrated that RA prevalence was 0.5-1% in Europe and North America [6]. According to statistics, RA was seen to be occurring predominantly in the native American-Indian populations, with the disease being more ubiquitous in Pima and Chippewa Indians, highlighting a prevalence of 5.3% and 6.8%, respectively [7,8]. The number of older adults with RA is rising as life expectancies rise globally. It is crucial since treatment objectives vary depending on the patient's age or the existence of infection risk factors, something that rheumatologists and primary care physicians should continuously evaluate; the lifetime risk of RA is 4% in women and 3% in men [1]. Conforming to the scientific data, RA is shown to be the product of an interaction between hereditary and environmental consequences, with the majority of risk factors gravitating to be gene polymorphisms (PTNP22), smoking, hormones, and positive family history [9,10]. RA predominantly affects the metacarpophalangeal, metatarsophalangeal, and proximal interphalangeal joints with subsequent involvement of the wrist and knee [11]. Clinically, RA presents with swelling, morning stiffness, and limited joint movement, with the development of immobility and painful deformities as a long-term sequel of the disease [11]. In consonance with the progressive nature of the disease, RA tends to involve the cervical spine and the temporomandibular joint along with severe systemic complications such as arrhythmias, septic infections, miliary tuberculosis, and mononeuritis multiplex associated with vasculitis [12]. Physical examination reveals joint tenderness, and the diagnosis is confirmed by the presence of rheumatoid factor and anti-cyclic citrullinated peptide antibody (anti-CCP Ab) [12]. Additional workup for RA includes determining levels of inflammatory markers such as C-reactive protein (CRP) and erythrocyte sedimentation rate (ESR) along with supplementary radiographic investigations and synovial fluid analysis [11-13]. Although disease-modifying antirheumatic drugs (DMARDs) are considered to be the first choice of treatment, medications such as non-steroidal anti-inflammatory drugs (NSAIDs) and glucocorticoids have been shown to alleviate the pain and inflammation associated with RA itself [14]. Biologics play a pivotal role in counteracting the inflammatory consequences of cytokines, thereby decreasing the severity of the disease in the long run [13]. They include chimeric monoclonal antibodies (Abs) directed against CD20, tumor necrosis factor (TNF)-alpha inhibitors, pegylated and humanized monoclonal Abs, interleukin (IL)-1 receptor antagonists, and anti-IL-6 receptor antagonists [14]. However, the role of biologics in managing RA is highly dependent on the patient profile and the response of the disease to previous therapy. This review article aims to explore the role of biologics in the treatment of RA in decreasing the severity and improving the prognostication of the disease.

## Review

Based on previous studies, cytokines play a crucial role in RA pathogenesis and a therapeutic application; therefore, it is foremost in the remedial aspect to restrain cytokines and small molecules. In commencing phase, prior studies conclude that B and T cells are triggered primarily by the release of cytokines that play a vital role in RA development.

Based on previous studies, cytokines have come to be identified as important factors that contribute to the release of B and T cells, thereby playing an essential role in the pathogenesis of the disease [15]. Pro-inflammatory cytokines such as TNF-alpha, IL-1, IL-6, 1L-12, and CTLA-4 induce joint destruction, promote pannus formation, and elicit extra-rheumatic manifestations causing widespread multi-organ damage to the body [16]. Self-tolerance and autoimmunity prevention tend to be jeopardized as the disease progresses due to a decline in the regulatory T-cells (T-reg) [15,16]. Since helper T cell (Th)-17 cells induce neutrophilic inflammation by the production of IL-17, IL-21, and IL-22, it becomes crucial to recognize that Th-17 cells hold immense importance in governing autoimmunity in comparison to Th-1 cells [15,16].

The course of the disease also witnesses an increase of retinoic acid receptor-related orphan nuclear receptor (ROR-c) and a decrease of FOXP3 expression due to transforming growth factor-beta (TGF-beta) and signal transducer and activator of transcription (STAT-3), which rely on the release of cytokines such IL-16 and IL-21. In contrast, when there is no inflammatory condition, ROR-c and FOxP3 are inhibited by T-reg cells, ultimately leading to a downfall in the expression of IL-17 and IL-21 cytokines [17,18].

**Figure 1:**
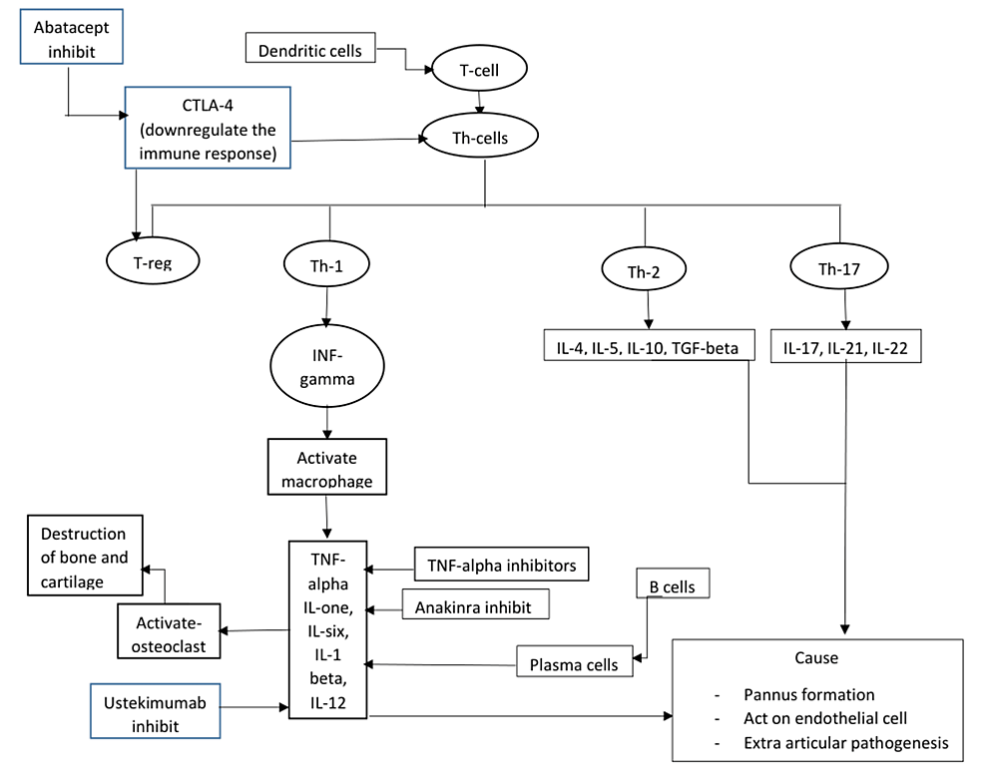
A summarized flowchart of biologics and cytokines IL, interleukin; TGF, transforming growth factor; Th, helper T cell; TNF, tumor necrosis factor; Treg, regulatory T cell Image credit: Jay P. Patel

TNF-alpha inhibitors such as etanercept, infliximab, adalimumab, certolizumab, and golimumab are known to have the longest safety data and remain the initial choice of treatment in patients requiring biologics; methotrexate (MTX) is the preferred choice of DMARD as a first-line agent, given its excellent efficacy in achieving remission or low disease activity (LDA) in 30% to 40% of patients [1]. In addition, IL inhibitors such as anakinra, rilonacept, canakinumab, tocilizumab, and ustekinumab exert influence on IL-1, IL-1 beta, IL-6, IL-12, and IL-23, and abatacept, which suppresses CTLA-4, has also been shown to have a persuasive action in RA pathogenesis [1]. The above discussion is summarized in a flowchart (Figure 1).

Role of TNF-alpha inhibitors

TNF-alpha inhibitors use has increased drastically over the last few decades. A study conducted by Radner and Aletaha stated that in the last few decades, the treatment of RA is revolutionized, as it is a specific action to restrain the disease activity, and also new drugs have been introduced in the market that has shown promising results, such as infliximab, adalimumab, certolizumab, abatacept, and golimumab [19].

According to a study by Radner and Aletaha, the introduction of TNF-alpha inhibitors and new-age drugs had been revolutionized the treatment of RA by restraining the disease activity and slowing the progression in the last few decades [19]. Although a large number of drugs have been sought to induce remission in RA, including TNF-alpha inhibitors, there is an uprising concern and debate regarding the discontinuation of the drugs once remission has been achieved [19]. The classification criteria for RA [1] are given in Table 1.

**Table 1:** Classification criteria of rheumatoid arthritis MCP, metacarpophalangeal; PIP, proximal interphalangeal; IP, interphalangeal; MTP, metatarsophalangeal; RF, rheumatoid factor; ACPA, anti-citrullinated peptide antibody; CCP, cyclic citrullinated peptide; ULN, upper limit of the normal range; CRP, C-reactive protein; ESR, erythrocyte sedimentation rate

		Score
Joint involvement	One large joint (shoulder, elbow, hip, knee, ankle)	0
	2-10 large joints	1
	1-3 small joints (MCP, PIP, thumb IP, 2 MTP, wrists)	2
	4-10 small joints	3
	>10 joints (at least one small joint)	5
Serology	Negative RF and negative ACPA	0
	Low-positive RF or low-positive anti-CCP antibodies (<3 times ULN)	2
	High-positive RF or high-positive anti-CCP 3 antibodies (>3 times ULN)	3
Acute-phase reactants	Normal CRP and normal ESR	0
	Abnormal CRP or abnormal ESR	1
Duration of symptoms	<6 weeks	0
	>6 weeks	1

In the study conducted by Radner and Aletaha, the first drug introduced in RA patients was infliximab [19]. With a half-life of 8 to 10 days, infliximab is known to be a chimeric monoclonal Ab that suppresses the cytokine activating the TNF receptor complex and is given through the intravenous (IV) route every four to eight weeks [20].

As we know, TNF-alpha is associated with systemic inflammation and acute phase reactions, producing CD4 + lymphocytes, macrophages, natural killer (NK) cells, neutrophils, etc. Infliximab has shown a high affinity toward TNF-alpha, which is responsible for various physiological responses such as increasing adhesion molecules to release, induction of pro-inflammatory cytokines, and inflating the migration of leukocytes from the blood vessels into tissue [20]. Infliximab has been shown to decline the physiological complications associated with TNF-alpha, which mainly include systemic inflammation, increasing adhesion molecules, inducing pro-inflammatory cytokines, inflating leukocyte migration into tissues, and the diffuse activation of acute phase reactions [20].

An open randomized controlled trial conducted by Bao et al. at the Shanghai Changzheng Hospital and Shanghai Guanghua Hospital in China from December 2008 to August 2010 recorded RA patients' responses after being infused with infliximab [21]. Patients between 18 and 65 years of age were chosen based on the fulfillment of the revised 1987 American Rheumatism Association criteria for the classification of RA along with the presence of active disease [21]. The patients meeting the eligibility criteria were enrolled based on four or more swollen joints and with the presence of at least one of the following: An ESR of >28 mm/h, a minimum CRP level of 10 mg/L, and morning stiffness lasting >45 minutes [21]. A total of 104 patients were enrolled in the open trial despite being on MTX, and the patients with significant infections and co-existing autoimmune disorders were successfully excluded from the study [21]. Among the 104 patients, 27 of them who did not respond to IL-1Ra were called “switchers,” and 51 of them who had never received biologics were called “naivers” [21]. These patients who were concurrently on MTX therapy were infused with infliximab and followed for 18 weeks. The rest 26 patients continued with MTX therapy and came to be known as “controls” [21]. After the 18th week, the American College of Rheumatology (ACR) 20 response was recorded in 56% of “switchers” and 61% of “naivers” compared to 23% of the “controls” [21]. To summarize the conclusions of the study, “switchers” and “naivers” showed better outcomes for the swollen joint count, tender joint count, pain associated with RA, and levels of ESR and CRP in comparison to “controls” themselves proving the effectiveness of infliximab in maintaining joint function and improving disease activity [21] (Table 1).

St Clair et al. scrutinized a three-year study in which 1,049 patients were enrolled with active disease and had not taken the drug in the past. In this study, the patients were divided into a group of 4:5:5 ratio, and the drugs given to the patients included MTX-3 mg/kg infliximab, MTX- infliximab 6 mg/kg, and MTX-placebo infusions [22]. The infliximab or placebo infusions were given at 0, two, six, and every eight weeks and after that through week 46, along with the MTX dose inclined to a level of 20 mg per week [22]. At week 54, the median percentage of American College of Rheumatology improvement (ACR-N) was recorded to be higher in groups with MTX-infliximab infusions as compared to groups who were given MTX alone [22]. In addition, the MTX-infliximab groups showed better radiological improvement and a drastic alleviation in joint function as compared to the MTX-placebo groups [22]. Health Assessment Questionnaire (HAQ) disability index scores also showed a higher rectification in MTX-infliximab groups than in the other group enrolled in the study [22]. To conclude the finding of the study, patients with a combination therapy of MTX-infliximab in the early stages of RA showed better physical, radiological, and functional benefits as compared to taking MTX alone [22] (Table 1).

One of the biologics that has revolutionized the treatment of RA in recent years is etanercept. Radnar et al. described it as a genetically engineered drug that is comprised of the extracellular domain of the TNF-alpha receptor and the Fc portion of immunoglobulin G (IgG) [19]. It has the shortest half-life of 3.5-5.5 days and is administered subcutaneously twice per week with a dose of 25 mg [19].

Combe et al. conducted a double-blinded randomized controlled trial over a duration of two years involving 260 patients with active RA, thereby establishing the significance of the disease [23].

Enrolment included patients aged 18 years or older with a disease duration of 20 years or less with active RA, following an ESR > 28 mm/h at the end of the first hour and a serum CRP level of >20 mg/L [23]. The efficacy of the patients was reviewed using ACR, Disease Activity Scores (DAS), and patient-reported outcomes (PROs) [23]. The patients were randomly assigned into three groups, and the drugs infused were etanercept (etanercept 25 mg by subcutaneous injection twice weekly plus placebo), sulfasalazine (sulfasalazine 2, 2.5, or 3 g daily plus placebo), or a combination (etanercept plus sulfasalazine) therapy [23]. The mean DAS scores that were recorded in participants were 5.1, 5.2, and 5.1 for etanercept (n = 103), etanercept plus sulfasalazine (n = 101), and sulfasalazine (n = 50), respectively [23]. A significant number of the patients who continued on sulfasalazine alone (68%) withdrew from the study as compared with those receiving etanercept, either as a combination (24%) or replacement (37%) therapy (p<0.001) [23]. Patients receiving etanercept or the dual therapy of etanercept and sulfasalazine showed a more incredible initial response as compared to the sulfasalazine alone: DAS scores reduced to 2.8, 2.5, and 4.5, respectively (p<0.05), ACR 20 response was recorded to be 67%, 77%, and 34% of patients, respectively (p<0.01), while the PRO responses were achieved as 76%, 78%, and 40% of patients, respectively (p<0.01) [23]. The study concluded the efficacy of RA management with etanercept or the combination of etanercept and sulfasalazine, thereby adopting the drug for the long-term management of the disease itself [23].

Iannone et al. conducted a retrospective study on patients who switched from infliximab to etanercept to prove the efficacy of the latter drug [24]. The study was conducted by gathering the data over a period of 24 weeks on 553 patients diagnosed with RA who were being treated with infliximab but were shifted to etanercept due to side effects. The assessment was conducted based on the ESR, CRP, and DAS computed on 44 joints, visual analog scale of pain (VAS), and HAQ every eight weeks [24]. The results came out at the end of the assessment and no significant difference between infliximab and the etanercept was recorded: DAS-44 scores of 2.7 and 1.9, respectively, HAQ scores of 0.75 and 0.75, respectively, ESR of 21 and 14, respectively, CRP of 0.5 and 0.3, respectively, and VAS scores of 40 and 24, respectively [24]. The study concluded that the withdrawn TNF-alpha inhibitor could be replaced with another TNF-alpha inhibitor and still maintain the clinical benefit of the management of RA itself [24].

Adalimumab is a monoclonal Ab of recombinant IgG that works opposite TNF-alpha, suppresses the cytokine-related inflammatory processes, is similar to the naturally occurring human IgG, and has a low immunogenic potential [25]. It activates NF-kappa B receptors on stromal and osteoblast cells and blocks bone and cartilage destruction [25].

In 2011, Machado et al. performed a systematic review and meta-analysis of randomized controlled trials to prove the safety and efficacy of the drug [26]. A total of 11 articles were included, and nine studies were considered with a total of 3,461 patients [26]. The drugs given to patients were adalimumab-MTX and placebo-MTX in a span of 24 to 104 weeks [26]. Patients with adalimumab-MTX showed better results with regard to efficacy and lowering radiological progression of the disease as compared to placebo-MTX alone [26].

Okano et al. performed a prospective randomized trial by administrating adalimumab drug to 68 RA patients over a period of three years, among which 29 patients successfully achieved LDA [27]. For the remaining patients, neither increase in dosage nor switching to another drug class showed a difference in LDA [27]. Therefore, it can be inferred that after achieving LDA in RA patients, thesame therapy can be continued to achieve better results for a longer duration [27].

Golimumab is a monoclonal Ab that can bind both soluble and transmembrane TNF, thereby preventing binding and activity of the TNF-alpha receptors, and is administered by subcutaneous injections (50 or 100 mg) every four weeks [28]. Smolen et al. assessed a multicenter, double-blinded, randomized study between February 21, 2006, and September 26, 2007, to evaluate the efficacy and safety of golimumab in RA patients who had received TNF-alpha inhibitors in the past [28]. A total of 461 patients with RA were enrolled from 82 sites over 10 countries, and the drugs given were placebo (n=155), 50 mg golimumab (n=153), and 100 mg golimumab (n=153) at an interval of every four weeks [28]. At the end of the 14th week, the assessment revealed an improvement of >20% in the ACR20 score [28]. At 16th week, patients who did not show this kind of benefit were switched to a different therapy such as from placebo to 50 mg golimumab or from 50 mg golimumab to 100 mg golimumab [28]. The drug safety was assessed between 1 and 16 weeks, and serious side effects were listed in 11 (7%) patients on placebo, eight (5%) patients on 50 mg golimumab, and four (3%) patients on 100 mg golimumab [28]. At the end of the study, golimumab was well tolerated, no malignant adverse effects were recorded, and disease reduction was more pronounced in the group of 100 mg golimumab compared to 50 mg golimumab and the placebo group [28].

The study by Smolen et al. was a multicenter, randomized, double-blind, placebo-controlled study over a duration of 160 weeks and involved patients with active RA, who reported four or more tender joints and four or more swollen joints in the past [29]. The patients were divided into three groups: group 1 was given a placebo, group 2 was given 50 mg golimumab, and group 3 was given 100 mg of golimumab in the form of subcutaneous injections every four weeks [29]. At the 24th week, group 1 was crossed over to golimumab 50 mg, group 2 continued with 50/100 mg, and group 3 proceeded with the same dose [29]. The patients were assessed from week 24 to week 100: ACR20 (≥20%) response and ≥0.25 units of HAQ improvement were retained in 70-73% and 75-81% of the patients, respectively [29]. At week 160, 59%, 65%, and 64% of patients had HAQ improvement ≥0.25 units, and 63%, 67%, and 57% of patients achieved ACR20 response in groups 1, 2, and 3, respectively [29]. Out of the 459 patients enrolled for treatment, 236 completed the therapy through week 160. The study concluded sustained improvement in the signs, symptoms, and physical functions of 57-67% of patients who took 50 mg and 100 mg of golimumab while discontinuing the previously used TNF-alpha antagonists [29]. Not only does the study manage to prove the efficacy of golimumab, but it also highlights the need for strict monitoring with regard to the long-term safety of the drug [29]. Summary of studies in Table 2 contains information on TNF-alpha inhibitors.

**Table 2:** Summary of included studies contains information on TNF-alpha inhibitors CRP, C-reactive protein; ESR, erythrocyte sedimentation rate; LDA, low disease activity; MTX, methotrexate; RA, rheumatoid arthritis

S. no.	Reference	Duration	Population	Drugs	Conclusion
1	Bao et al. [21]	December 2008 to August 2010	104 patients	Infliximab, MTX, placebo	Infliximab therapy showed a better performance in the “switchers” and “naivers” compared to “controls” with regard to improvement in joint swelling, ESR, and CRP.
2	St Clair et al. [22]	3 years	1,049 patients	Infliximab-MTX MTX-placebo	Infliximab-MTX showed a better physical, functional, and radiological improvement in comparison to patients with MTX-placebo.
3	Combe et al. [23]	2 years	260 patients	Etanercept sulfasalazine and in combination	Etanercept alone and etanercept in combination with sulfasalazine showed an impressive controllable achievement as compared to sulfasalazine alone.
4	Iannone et al. [24]	Data gathered over a span of 24 weeks	553 patients	Etanercept efficacy	The drug showed great efficacy in maintaining and improving the disease activity.
5	Machado et al. [26]	24 weeks	3,461 patients through 11 articles including a studies	Adalimumab-MTX and placebo-MTX	Adalimumab given every two weeks proved to be more effective and well-tolerated in the long term.
6	OKano et al. [27]	3 years	68 patients	Adalimumab	Adalimumab treatment could still be continued as the same therapy after achieving LDA in the majority of patients.
7	Smolen et al. [28]	February 2006 to September 2007	461 patients	Golimumab, placebo	Golimumab was well tolerated with no malignant adverse effects and showed a greater reduction in the disease as compared to the placebo.
8	Josef Smolen al. [29]	160 week	459 patients	Golimumab, placebo	Golimumab showed 57-60% improvement with regard to RA.

Based on observational and interventional types of studies, research was conducted over a duration of one year in a sample population of 346 RA patients by combining 20 research conducted in the past [30]. The categories were based on drugs being allocated to each patient, with 61 patients receiving infliximab, 122 receiving adalimumab, 82 receiving etanercept, and 81 receiving anyone from the above three with no specific data to present [30]. Ursini et al. reviewed these studies and concluded that the TNF-alpha inhibitors given in patients showed an improvement in the cardiovascular system (CVS) and endothelial function with a decrease in arterial wall stiffness and atherosclerosis. A meticulous study was difficult to conduct, and hence it was concluded that a larger sample population and an extended observation period would be required to mitigate the limitations and obtain more conclusive results [30].

Certolizumab is a humanized monovalent Fab Ab fragment linked to polyethylene glycol (PEG) and tends to have a different mechanism of action as compared to other TNF inhibitors [19]. The PEG portion is a bulky hydrophilic inert molecule, which increases the plasma half-life of the drug (estimated to be two weeks), and the dose recommended for adults are 400 mg (given as two subcutaneous injections of 200 mg) initially and at weeks 2 and 4, followed by 200 mg every other week [19].

Role of IL inhibitors

Anakinra is an IL-1one inhibitor and plays a measure role in augmenting the blockade of IL receptors and modeling the progression of the disease [31]. Nikfar et al. evaluated the drug efficiency and safety by studying clinical trials and extension studies through databases such as PubMed, Scopus, and Web of Science till November 2017 [31]. About 10 studies were included in the review, and the ACR20 was the efficacy outcome measure [31]. Although there was a 34% risk of treatment withdrawal associated with the therapy, the patients receiving anakinra showed a 42% likelihood of an ACR20 response, including a significant decline in ESR and HAQ scores [31].

Cohen et al. performed a multicenter, double-blinded, randomized, placebo-controlled trial, which included 506 RA patients who were already being treated with MTX [32]. An addition of a placebo and anakinra was infused, and a monthly assessment was conducted to evaluate the side effects and the progression of the disease over a period of six months [32]. Patients treated with anakinra showed impressive results with ACR50 (17% vs 8%; p<0.01), ACR20 (38% vs 22%; p<0.001), and ACR70 (6% vs 2%; p<0.05). Not only were the responses toward anakinra rapid and effective, with improved radiological and functional findings in RA, but also the drug provided favorable outcomes in inflammatory parameters such as ESR and CRP [32].

IL-6 is a pro-inflammatory cytokine that binds to receptors and activates intracellular signaling pathways that affect the acute phase response, cytokine production, and osteoclast activation, and play a detrimental role in affecting joint inflammation and the progression of the disease [33]. Tocilizumab is a humanized monoclonal Ab that is directed against the soluble and membrane forms of IL-6 receptors, suppresses cytokine production, and is associated with increased risk of pancytopenia, infection, and atherosclerosis [33].

Emery et al. conducted a multicenter randomized placebo-controlled trial with tocilizumab which included 499 patients, who were assigned a dose of 4 mg/kg or 8 mg/kg or placebo intravenously every four weeks along with MTX over a span of 24 weeks [33]. At the end of 24 weeks, ACR20 recorded for the patients was 50% (8 mg/kg), 30.4% (4 mg/kg), and 10.1% (control group), correspondingly (p<0.001) [33]. The adverse reactions associated with the drug included rash, headaches, and gastrointestinal problems, and the incidence was shown to be more severe in the controls as compared to the patients on tocilizumab [33].

Other biologics

Ustekinumab is a human monoclonal Ab that exerts an effect on IL-12 and IL-23 by inhibiting cytokines to suppress the disease activity [1]. Abatacept is a fusion protein that has shown a promising outcome in reducing the disease activity and in radiographic advancement by inhibiting co-stimulation of T-cells and improving the functional disability associated with RA [1]. It is used mostly in combination with other therapies because of its slower onset action and has shown to have an increased risk of infection but is usually well tolerated otherwise [1]. Rituximab is a chimeric monoclonal Ab directed against CD20, is expressed mostly by mature B-lymphocytes, and is approved for the management of refractory RA in combination with MTX where it shows better tolerance with the seropositive disease as it has been associated with a higher risk of infection such as progressive multifocal leukoencephalopathy [1].

Perez-Sanchez et al. conducted a cohort study over three months in Spain where 75 RA patients and 90 healthy control patients were enrolled in the study [34]. Along with this, another study was conducted with 16 patients for researching the effects of rituximab (CD20 inhibitor) over the immune system and its role in suppressing the progression of the disease. The data aggregated at the end of the study highlighted the efficacy of rituximab in declining the percentage of B-cells and cytokines in RA [34]. The study was assessed based on B-cells and pro-inflammatory cytokines, while the test used in the evaluation were reverse transcription polymerase chain reaction (RT-PCR) and flow cytometry, which provided enough results to support that early therapy of rituximab resets the cytokines and maintains the balance in the immune system and vascular wall [34].

Studies were reviewed to understand the occurrence of risk of infection with biologics [35]. Galloway et al. examined the data from the British Society for Rheumatology Biologics Register (BSRBR) comparing the risk of serious infections in 11,798 TNF-alpha inhibitor-treated patients. Of these, 1,808 patients had at least one serious infection, with incidence rates of 42/1,000 patient-years of follow-up [35].

This study shows that the risk of serious infections with anti-TNF-alpha was increased in the first six months of initiating therapy for RA, but gradually declined after a span of 24-36 months [35]. A prospective study was conducted by van Dartel et al. over a duration of five years to follow up patients with anti-TNF-alpha and observe the risk of infection by using the Dutch Rheumatoid Arthritis Monitoring (DREAM) registry focusing on comparisons of patients starting one of the three anti-TNF-alpha inhibitor (etanercept, infliximab, adalimumab) [36]. The patients were initially assessed for any co-morbid condition and were evaluated during regular follow-ups every two months up to two years and after that every six months. After five years of study, it was concluded that the incidence of the infection in RA per 100 patients was 2.61 (95% CI: 2.21 to 3.00) for adalimumab, 1.66 (95% CI: 1.09 to 2.23) for etanercept, and 3.86 (95% CI: 3.33 to 4.40) for infliximab. Thus, the risk of infection associated with etanercept was lower than with infliximab and adalimumab [36]. The infectious complication of TNF-alpha inhibitors is bacterial (tuberculosis reactivation, nocardiosis, listeriosis), fungal (invasive candidiasis, pneumocystosis), and viral (hepatitis-B reactivation, herpes zoster reactivation), along with induction of lupus nephritis [36] (Table 3).

**Table 3:** Risk of serious infections with biologics

Infection	
Bacterial	Tuberculosis reactivation
	Nocardiosis
	Listeriosis
Fungal	Invasive candidiasis
	Pneumocystosis
Viral	Hepatitis-B reactivation
	Herpes zoster reactivation

Some studies suggested the reactivation of cytomegalovirus (CMV) with TNF-alpha drugs when combined with immunosuppressive medications [37]. Reactivation of Epstein-Barr virus (EBV) and other herpes viruses has also been disclosed rarely with abatacept, but it is unclear if these cases are due to previous or concomitant immunosuppression, as another study also illustrates that long-term use of abatacept does not affect immunological control of EBV infection. In the long run, the drugs infliximab and adalimumab can develop anti-drug Ab [38].

Strengths and limitations

Although RA is a complex systemic disease and has multiple etiologies, our review targets to cover the inhibition of cytokine action on bone degradation by biological drugs. However, this study does not address all the biologics because of the limited studies available; therefore, more studies need to be conducted to fill the gap.

## Conclusions

RA is a complex systemic disease characterized by persistent synovitis, autoantibodies, and a wide variety of extra-articular manifestations. The risk factors involved in RA pathogenesis are predominantly genetic and environmental and play an important role in triggering cytokine cascades that result in joint destruction due to pannus formation. In recent decades, biologics have been shown to block cytokine action on a large scale and significantly improve joint function and quality of life. This study successfully identifies the role of biologics in changing the path of disease, reducing drug dependence, preventing infections (bacterial, viral, and fungal), and alleviating adverse effects associated with the medications. This article benefits students and physicians by reviewing the pathogenesis of biological drugs such as TNF-alpha, IL, and CTLA-4 inhibitors. The clinical implication of this review is to identify the RA patient through the classification criteria and to imply the appropriate biological drugs. This article serves as a means to overcome the challenges in RA by providing different approaches to tackle the disease through biological drugs and assess the disease from time to time by ESR, CRP, rheumatoid factor, anti-CCP, and so on. Lastly, more in-depth research studies may be required, and a more comprehensive approach is necessary to help various issues in RA management.
